# Play the Pain: A Digital Strategy for Play-Oriented Research and Action

**DOI:** 10.3389/fpsyt.2021.746477

**Published:** 2021-12-15

**Authors:** Najmeh Khalili-Mahani, Eileen Holowka, Sandra Woods, Rilla Khaled, Mathieu Roy, Myrna Lashley, Tristan Glatard, Janis Timm-Bottos, Albert Dahan, Marieke Niesters, Richard B. Hovey, Bart Simon, Laurence J. Kirmayer

**Affiliations:** ^1^McGill Centre for Integrative Neuroscience, Montreal Neurological Institute, McGill University, Montreal, QC, Canada; ^2^Division of Social & Transcultural Psychiatry, McGill University, Montreal, QC, Canada; ^3^Culture and Mental Health Research Unit, Lady Davis Institute, Jewish General Hospital, Montreal, QC, Canada; ^4^Technoculture, Arts and Game Centre, Milieux Institute for Art, Culture and Technology, Concordia University, Montreal, QC, Canada; ^5^Patient Partner, Montreal, QC, Canada; ^6^Department of Psychology, McGill University, Montreal, QC, Canada; ^7^Department of Computer Science, Concordia University, Montreal, QC, Canada; ^8^PERFORM Centre, Concordia University, Montreal, QC, Canada; ^9^Department of Creative Art Therapies, Concordia University, Montreal, QC, Canada; ^10^Department of Anesthesiology, Leiden University Medical Centre, Leiden University, Leiden, Netherlands; ^11^Faculty of Dentistry, McGill University, Montreal, QC, Canada; ^12^Department of Sociology, Concordia University, Montreal, QC, Canada

**Keywords:** chronic pain, personalized medicine, citizen labs, stigma & discrimination, digital health, serious games (SGs), big-data, play

## Abstract

The value of understanding patients' illness experience and social contexts for advancing medicine and clinical care is widely acknowledged. However, methodologies for rigorous and inclusive data gathering and integrative analysis of biomedical, cultural, and social factors are limited. In this paper, we propose a digital strategy for large-scale qualitative health research, using *play* (as a state of being, a communication mode or context, and a set of imaginative, expressive, and game-like activities) as a research method for recursive learning and action planning. Our proposal builds on Gregory Bateson's cybernetic approach to knowledge production. Using chronic pain as an example, we show how pragmatic, structural and cultural constraints that define the relationship of patients to the healthcare system can give rise to conflicted messaging that impedes inclusive health research. We then review existing literature to illustrate how different types of play including games, chatbots, virtual worlds, and creative art making can contribute to research in chronic pain. Inspired by Frederick Steier's application of Bateson's theory to designing a science museum, we propose DiSPORA (Digital Strategy for Play-Oriented Research and Action), a virtual citizen science laboratory which provides a framework for delivering health information, tools for play-based experimentation, and data collection capacity, but is flexible in allowing participants to choose the mode and the extent of their interaction. Combined with other data management platforms used in epidemiological studies of neuropsychiatric illness, DiSPORA offers a tool for large-scale qualitative research, digital phenotyping, and advancing personalized medicine.

## Introduction

### Chronic Pain: The Limits of Biomedical Models

Chronic persistent pain (CP) is a leading cause of disability worldwide (1–5). Medical science has had limited success in treating many forms of CP and there is increasing recognition that responding to CP requires more than purely biomedical models (6, 7). Contemporary approaches recognize that the sensory processes of pain are modulated by top-down influences including expectations, emotions, and cultural mediated meanings (8). Melzack's neuromatrix pain theory, for example, considers that variations in nociceptive sensitivity may arise from genetic predispositions (9), as well as from a cascade of emotional and cognitive factors that modulate the sensory neural signaling resulting in different experiences among individuals even when they experience similar injury, and for the same individual in different contexts (6). Psychological stress affects pain perception (6, 10, 11). Sociopolitical, economic and cultural stressors do too (12–14). As Craig's Social Communication Model of Pain emphasizes, because humans must adapt to complex social environments, social contexts impact on how pain is experienced, and thus cognitive and social processes and contexts must be considered in understanding patients' own perceptions and behaviors in presence of pain (15). The neuromatrix and communication models can be integrated and extended by recognizing that bodily experiences and social contexts provide metaphors for thinking about and expressing pain experience—and can reshape that experience (16). Pain experience is mediated, elaborated and communicated through cultural models which need to be studied with qualitative methods (17–19).

Pain is inherently a subjective experience and the quality of communication between patient and clinician is crucial for assessment and treatment. Communicating effectively about one's pain in clinical settings is challenging (20). Social factors, including power imbalances inherent in healthcare systems (in which both patient and physician may assume that doctors know better and their time is too valuable to waste), can distort effective communication and silence the patient (21–27). Miscommunication can lead to inaccurate or incomplete assessment, limit patients' access to resources for pain control, coping, and prevention and impede community support and medical care (28). The failure to adequately assess and track patients' experience of pain contributes to disparities in care and may impede progress in the development of innovative interventions.

Partnerships between people with lived experience of pain and researchers are recognized as vital for advancing knowledge and practice in CP care (29–33). However, the experience of CP remains extremely challenging to communicate, quantify, and explain to others (34, 35). Unlike many chronic health conditions (such as hypertension), there are no reliable objective measures or biomarkers for pain, hence patients' self-reports are crucial. Even standardized psychometric instruments may miss patients with clinically important pain (36). The framework we describe in this paper aims to provide an innovative approach to pain experience in research and clinical settings by using digital technology to facilitate playful exploration and reflection.

### Integrating Context and Experience in Pain Research

Given the diverse cultural contexts, constructs, and belief systems that shape how individuals communicate distress and seek care, it is difficult to quantify individual differences in pain experience (17, 20, 37). By extension, it is difficult to offer personalized care without an understanding of the social-cultural contexts or ecologies in which individual experience is embedded and from which it draws meaning and consequence. These contexts have physical, geographical, ideological, linguistic, political, and historical dimensions that need to be studied qualitatively. Qualitative analysis can inform the development of quantitative measures that more accurately capture the lived experience and consequences of pain and that can be used in large-scale studies. However, qualitative research is labor intensive even with small samples and hence cannot insure representativeness or generalizability of results. Large samples are needed to canvas the range of experiences, establish generalizability, and better understand the interaction of multiple variables.

Large-scale qualitative research poses methodological challenges related to: gathering data rigorously (38); creating a space for research partners to communicate and actively collaborate (39); ensuring inclusivity and equitable opportunities for shared learning through mutual respect; and disseminating knowledge through effective action (40). Faced with these challenges, biomedical research (which aims to produce “hard evidence” through rigorous hypothesis testing) may limit engagement with modes of knowledge creation and practice that do not fit easily with its epistemic culture (21, 22) and opt for methods that elide patient experience (41).

### The Promise of Information and Communication Technologies (ICT) for Integrative Pain Research

ICTs are becoming widely used tools for research and clinical data collection (42). They allow incorporating social media, biosensing, and artificial intelligence in the collection and analysis of health data (43), thus increasing efficiency in the accumulation and analysis of large datasets for digital phenotyping (44) in fields as diverse as behavioral neuroscience and computational sociology. Given the global penetration of smart mobile technologies (78% in 2020), ICTs make it possible to collect ecological data, disseminate information, and offer digital interventions remotely (45). For example, a recent systemic meta-review by Finucane et al. found that interventions such as videoconferencing, interactive social media and weblogs, educational websites, and high-fidelity simulators targeting professionals for retraining were feasible and potentially impactful tools for reducing costs, improving patient care, facilitating communication and information sharing, as well as for data collection and decision-making support in palliative care (46).

Although often used to collect quantitative data, ICTs can readily be adapted to obtain rich qualitative data and configured in ways that allow the interaction essential for more participatory health research. Several approaches to deploying ICTs in health research are currently being tested. Initiatives such as “*Saskatchewan, let's move and map our activity”* used citizen science to study active living, with a mobile app incorporating various tools from location monitoring to questionnaires in order to identify factors that motivate individuals to be physically active in different seasons (26). Initiatives such as *PatientsLikeMe* serve as virtual science laboratories where, while conducting *N* = 1 self-trials (47), participants also provide support to each other and exchange knowledge by sharing personal experiences as well as biological samples (48). Further, recent advances in data science demonstrate that social media can be important platforms for data collection and community knowledge creation (49). For example, the impact of Twitter interactions on public health was extensively studied during the COVID-19 pandemic (50–52). Massive multiplayer online games (MMOG), or role playing games (RPG) are other examples of ICTs that have been used for interactive research data collection (53).

### Shortcomings of Current ICTs for Qualitative Research

Large-scale qualitative research among culturally, geographically, and linguistically heterogenous populations is challenging (38, 39). ICTs are readily deployed to accumulate large datasets and one simple application of ICTs in mixed-methods research is to facilitate survey studies that lead to the creation and validation of reliable instruments for quantitative research (45). Alternatively, passive data generation (through surveillance and monitoring), and data-mining algorithms can try to uncover patterns (and predict outcomes). However, passive data collection methods often overlook the specific contexts or circumstances that define an individual's agency, subjectivity, and ability to express their priorities and concerns (54, 55). Neither surveys nor surveillance systems (through biosensors or trackers) solicit participants' opinions or knowledge; they therefore invite criticism on the grounds of ecological validity as well as research ethics (56–58).

For these and other reasons, if data mining is to advance healthcare, there is a need to integrate the ethical principles and processes of participatory health research (PHR), which involves the systematic engagement of patients and other stakeholders in the process of initiating, designing and interpreting research to develop actionable solutions (59, 60). To achieve this, ICTs must go beyond passive data mobilization to create the conditions for patient-oriented and person-centered research that is informed and guided by individuals' experiences (26, 61, 62).

### Research Approach

In the past 20 years, researchers have created numerous cohorts with neuropsychological epidemiological data to discover the causes and correlates of various brain-related disorders (63–66). Vast resources have been dedicated to data mining from biological specimens, extensive arrays of psychometric and physiological measures, and various types of radiological data (67, 68). However, active engagement of stakeholders and research participants at every stage of project development from its earliest conception, through design, data collection and interpretation, to translation into policy and practice can improve the relevance, quality and impact of health research (40, 69–80).

Given this context, we aimed to explore how ICTs could be used to add capacity to integrate qualitative data with standard quantitative research methods used in epidemiological studies of brain-related disorders. Our approach was inspired by emerging research that has shown that play with digital media is a promising strategy for conducting integrative neuropsychological research (81–84).

Our long-term goal is to use technology to diversify and democratize experimentation and participatory knowledge creation in health research. Chronic pain is an important focus for this work for several reasons: Pain is a debilitating physical condition that is universally experienced (so much so that there are now machine learning algorithms to detect pain experience from facial expressions or postural patterns) (85); but the causes of the chronicity of pain remain poorly understood and clinical assessment and care do not adequately incorporate patients' experience and life contexts.

We brought together an interdisciplinary team of clinicians, social scientists, bioethicists, designers, and computer scientists, with support from an intersectoral funding opportunity in the Province of Quebec (Canada), to develop an innovative ICT framework for play-oriented pain research. We held a 2-day public workshop in October 2019, bringing together research team members, clinicians, and individuals with lived experience of chronic pain (86, 87). The workshop was held at Concordia University's 4TH SPACE, an open gallery designed to showcase research and seek engagement from the public who were invited with the following message: “*We are scientists, artists, therapists. We work to advance medicine through communication, community, creativity. Can we study pain by shedding on it the light of play?”* Discussions were stimulated by playful activities that were facilitated by creative arts therapists, as well as by presentations from neuropsychology students on non-pharmacological pain research (music, movement, yoga), and performances by artists (theater, poetry). Two questions were the focus of these discussions:

- What strategies can capture the psychosocial and cultural mediators of chronic pain?
- What strategies can increase the opportunities of co-learning and co-creation among medical professionals, researchers, and patients who deal with chronic pain?

To answer these questions, we built on the workshop findings by reviewing the relevant literatures and iteratively developing a framework through discussions among team members. The approach presented in this paper is based on our identification of pragmatic, structural and cultural conflicts that need to be addressed in designing health-related ICTs. In this paper, we propose a theory-based approach that can guide the design of such applications.

The theoretical perspectives we draw from include neuromatrix pain theory (9), pain communication theory (15), cultural somatization theory (88), and person-centered research (23, 32, 37). We apply Gregory Bateson's cybernetic theory of self to illustrate how play (as a state of mind, as a mode of creation and communication, and as a structured, yet flexible framework for individual and group exploration) can provide a critical tool to advance mind-body research. The framework we propose aims to use digital media and game-like activities to engage people in exploring the quality, parameters, and modulators of their pain experience. Using modes of play can afford people some distance from the suffering associated with their health problem, while mobilizing both coping strategies and social connection to others. This theoretical framework has evolved in tandem with development of a limited prototype in the form of a bilingual mobile phone application, *PlayThePain* (86).

The paper is structured in three parts: First, we outline a theoretical framework for play as a tool for pain research; we then present a working definition of play, illustrated with examples of different types of play-oriented activities that can be implemented in a digital framework for pain research; finally, we provide a critical overview of how digital playgrounds can be implemented to be maximally inclusive and adaptable.

## Theory: A Cybernetic Approach to Studying Pain By Playing

### Gregory Bateson's Steps to an Ecology of Mind

Gregory Bateson was an anthropologist and interdisciplinary biological epistemologist whose work focused on the comparative analysis of relationships and interactions in human systems, extended to the relationship between ideas and the circumstances that determined the survival or extinction of cultural or natural subsystems. The systemic, interactional, ecosocial view that Bateson championed provides an integrative framework for advancing transdisciplinary research (89, 90).

**Figure 1:**
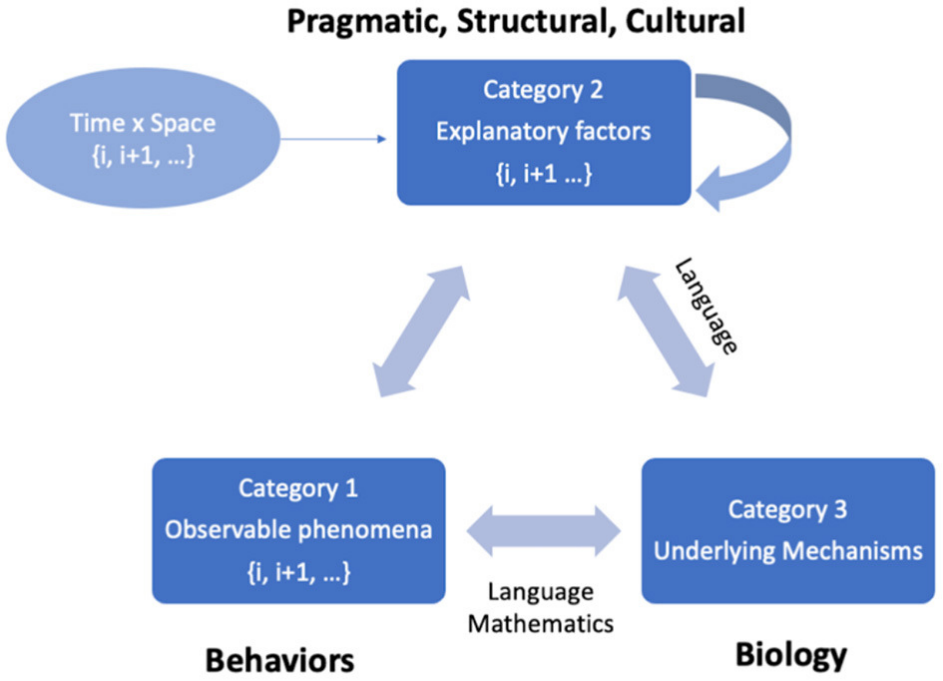
Bateson's cybernetic model of knowledge production. In neuropsychological research, the primary aim is to understand underlying mechanisms that give rise to an observable phenomenon. The relation between underlying mechanisms and the observable phenomena is mediated over time by various explanatory variables that may be interpreted flexibly within given contexts. Explanatory variables are influenced by various pragmatic, structural and cultural constraints and are prone to conflicts (including double binds) which involve recursive looping processes that must be studied over time and across contexts. The knowledge that emerges in this cybernetic model is often language dependent; however, if concepts are operationalized in measurable ways and sufficient data are collected, certain aspects can be represented by formal mathematical models.

In his collected papers, *Steps to An Ecology of Mind* (1972), Bateson laid the foundation of his cybernetic methodology—a recursive, simulation-based, experimental process that examines the emergence of meaning by observing variations in relationships, interactions, and feedback mechanisms between existing and emerging factors in a given system (91). His basic model of mind-body research distinguished three categories of knowledge, which can be applied to the study of chronic pain (Figure 1):

1. *Observable phenomena* (e.g., the manifest behaviors of an organism, such as avoiding painful stimuli or inability to move due to pain);
2. *Explanatory constructs* (e.g., self, motivation, anxiety, coping strategy, resilience); and
3. *Underlying mechanisms*, which either describe the processes involved in specific situations (e.g., prevalence of post-surgical pain) or common across varied contexts (e.g., the nociceptive pathways or circuits of the nervous system).

Our understanding of the relationship between observations and underlying mechanisms is mediated by a complex set of explanatory factors (Category 2) which have different degrees of pragmatic, structural, behavioral, and social-cultural constraints.

Because explanatory constructs are communicated through language (using personal and local cultural models and metaphors) and are employed in self-understanding and social presentations, they are susceptible to becoming entangled in what Bateson termed *double binds* (92, 93). Double binds arise in conditions where:

1. Two or more individuals are involved in an intense relationship with a high (physical or psychological) survival value for at least one of them (e.g., mother and child; physician and patient);
2. In this relationship, messages are regularly given that, at one level of communication, assert something, but at another level negate or conflict with this assertion (e.g., the mother yells at the child that yelling is bad; a physician who prescribed the narcotics to a patient stops providing the prescription because he fears the patient will become an addict);
3. The messaging implies a form of punishment or cost (e.g., the mother yells at the child that if they yell, they will be punished; the physician responds negatively to the patient who persists in requesting opioid medication);
4. Those in the relationship cannot escape the relationship, nor are they allowed or able to comment on it (e.g., the authority of mother over child; the power imbalance between physician and patient). (Further examples are provided in Table 1).

**Table 1:** Examples of double binds in chronic pain communication.

**Components of a double bind**	**Examples related to pain research and clinical care**
Intense relationship between two individuals that affects survival of at least one.	• The goal of the physician-patient relationship is the health and well-being the patient, but the biomedical priorities are usually treatment of underlying disease, symptom reduction, and improvement in functional status. • Patients rely on the physician for appropriate treatment, but the risks of drug over- or under-prescription or misuse pose challenges to the physician's sense of competence and professional reputation. • Individual care is embedded in larger societal issues related to disability and substance use. The government is responsible, or is seen as being accountable, for the health and well-being of the population.
Conflictual messaging	• Pain is a biological reality, but there are not yet accurate biomarkers for pain; hence its assessment and monitoring depend crucially on subjective report. • Medical practice aims to be evidence-based, but the evidence base is limited; pharmacological interventions are not always effective and many other forms of treatment remain untested. • Opioids are an important resource for pain management, but carry major risks for side-effects, and can impair functioning or exacerbate pain sensitivity over time.
Messaging that implies a cost or punishment	• If pain cannot be assessed objectively, then the patient who reports persistent pain may be viewed as exaggerating, amplifying, malingering or drug-seeking. • If patients' pain is not “real,” then they should be denied opioid prescriptions. • If physicians employ alternative treatments that are not backed by research evidence, they may be accused of quackery or malpractice.
The parties are not allowed to escape the interaction with each other or comment on the larger frame that confines them	• Patients are dependent on the biomedical health care system for authoritative treatment and for legitimacy in relation to disability, insurance, and compensation systems. • There are limited resources available for coping with chronic conditions. These are not equally accessible to different segments of society. • Individuals from particular cultural communities or backgrounds may have commitments to particular modes of treatment or coping that go beyond the focus of biomedicine and which they are not willing to set aside. • Professionals trained in evidence-based biomedicine may resign from providing care to patients who do not follow their medical advice but this raises ethical issues.

Bateson suggested that such double binds could lead to serious mental health problems, particularly schizophrenia. Subsequent research has not borne this out—double binds are ubiquitous and not uniquely associated with psychosis, indeed they can also motivate creativity (94)—but there is no doubt that double-bind situations are stressful and can erode trust and collaboration in health care settings (27, 95–99). Bateson suggested two strategies for overcoming double binds: (i) meta-communication (i.e., talking about the difficulty of talking) (94, 100); and (ii) collecting large amounts of data to identify the regularities behind cultural variation and to reveal (and momentarily step outside) the taken-for-granted frameworks that create double-bind situations [(91), p. 161]. Bateson also suggested that play serves as a tool for meta-communication, making it possible to simulate double binds and re-negotiate relationships through fictive conflicts [(91), p. 170–193].

### Play as Context and Method

In Western culture, “play” has been an object of theoretical reflection at least since Erasmus who argued that play is an existential necessity because it helps humans confront the inevitability of aging and death by becoming forgetful and carefree like children (101). Freud considered play a creative, adaptive, and therapeutic activity that generates pleasure by releasing tension (102, 103). According to Piaget, the pleasure of play is an important motivational asset, critical to behavioral and emotional development by promoting thinking, imagining, pretending, remembering, guessing, hoping, redoing, and working through problems (104). This developmental role is obvious in childhood but, in different forms, extends across the human lifespan. Huizinga argued that play is a precursor to the creation of culture, based on numerous examples showing that play is a uniquely human tendency to create imaginative aesthetics and rituals (religion, poetry, architecture, etc.), that give different meanings to the acts of satisfying biological needs (e.g., shelter, food, safety) (105).

Formal definitions of play have tried to operationalize it as a specific state of mind, mode of action, or form of communication. Play is “state of being active, operative, unimpeded in the logic of movement or the scope for action” (106). But play can also be defined by specific set of rules that structure actions in ways that may not have an obvious utilitarian function, such as in games or performances (on stage, in music, or sports). Play can engage with contradictory or ambiguous aspects of experience and explore new meanings and possibilities through performance. This can occur in solitary activities, small groups, or larger carnivalesque forms of play that allow individuals and communities to move beyond the dominant norms (frames) and interpretations of an experience (107).

For Bateson, all the characteristics of play (as state of mind, mode of action, and framework for communication) make play an experimental space for *recursive learning* in which participants learn by simulation and iterative interpretation and evaluation of outcomes without real-world costs or consequences. The paradoxical logic of play (for example, two people preparing to “fight” without intention to fight) allows simulating a double bind in which reality and fantasy co-exist, and relationships can be negotiated. By framing the meaning of actions as “just play,” the play situation then provides opportunities for meta-communication—reflecting on the rules and regulations that usually govern social interaction and experience [(91), p. 170–193]. Indeed, the anthropologist Sutton-Smith suggests the rhetorical uses and ambiguity of play (e.g., children's play, games and gambling, role playing, creative play, leisure and relaxation, or joking and foolishness) make it a useful tool for ethnographic research into how cultures construct meaning (108).

The message “this is play” creates opportunities for imaginative exploration in the safety of a context or situation defined as “as if” —that is, as outside the usual norms and structures of consequential action. Because the stakes in play are not consequential, the frame-flexibility can be modified. Rules of play, for example, can be negotiated to create new imaginative scenarios that provide opportunities for learning by observing causal links (through cycles of sensorimotor action) and by discovering tensions and possibilities in extended images, metaphors, and narratives. Through these experimental engagements with play, we can document how people with different pragmatic, structural and cultural resources, and constraints learn to adopt new behaviors and adapt to challenging experiences [(91), p. 170].

### Applying the Cybernetic Model to Pain Research

Applying Bateson's cybernetic model (Figure 1) to the phenomenon of pain (9), a person's descriptions and expressions of pain (verbal, facial, bodily gestures, functional, or behavioral) are the observable phenomena, and the underlying mechanisms are the cognitive, physiological, and neural processing that subserve pain experience. Top-down processes of cognition, affect regulation, and expectation influence the activity of sensory pathways and central information processing (19, 109). These links are partly hardwired, partly learned, and also influenced by the ways that individuals interpret and narrate their experience. Communicative interactions between patients and others in the social world, including care providers, influence the way the observable phenomena and underlying mechanisms are linked (15). Social-cultural differences in embodied experience and metaphors for pain further mediate the relationship between the observed phenomena and mechanistic processes (17, 19, 37).

**Figure 2:**
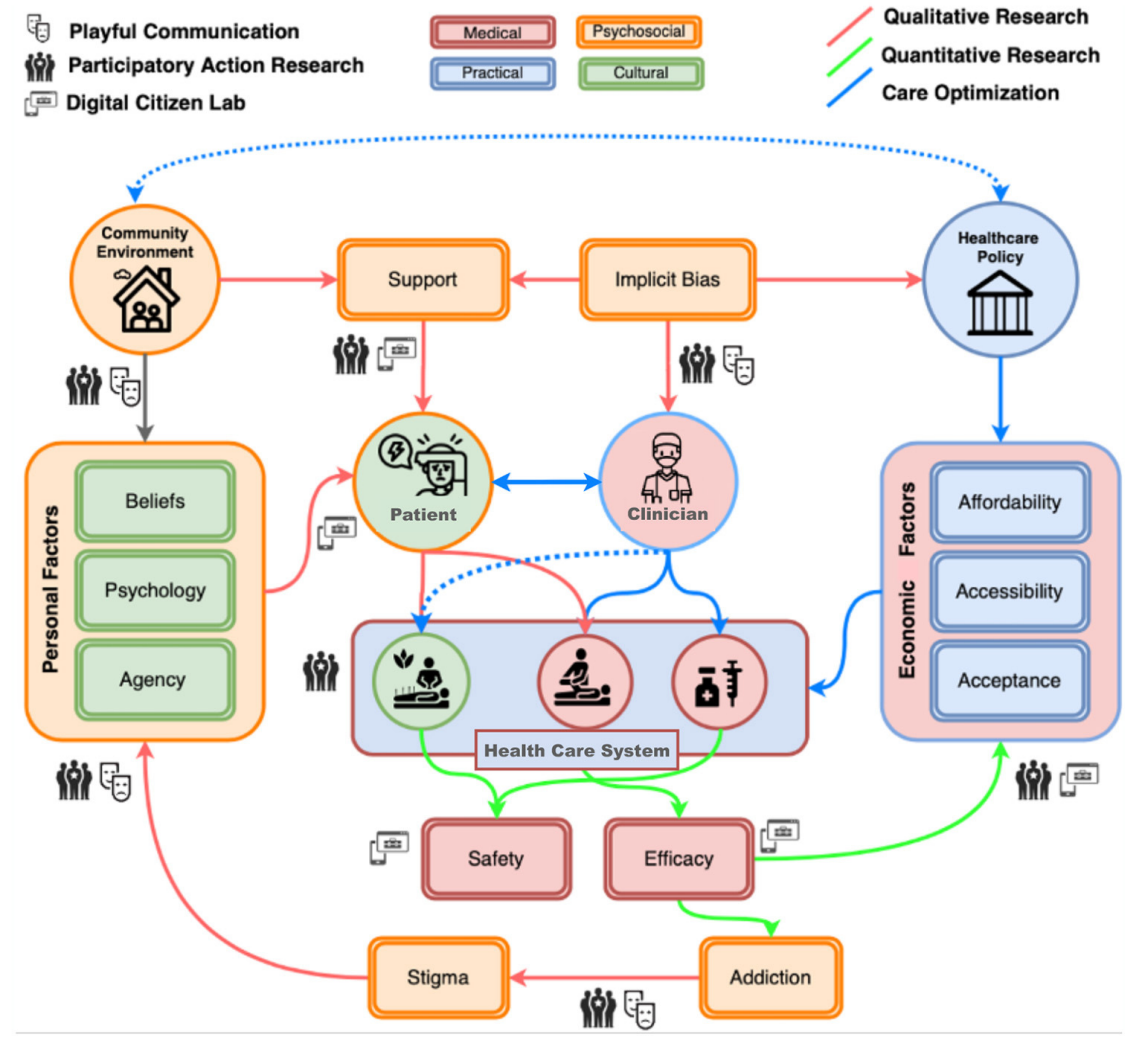
An extended cybernetic model for pain research. This diagram represents potential sites for data collection in the system of relationships between factors that govern the interaction of an individual patient and the health care system. Variables that relate to patients (indicated in green) include personal experiences and expressions of pain, as well as individual beliefs, psychological traits, and behaviors that express patients' agency, e.g., their treatment preferences and choices. Variables that relate to medical professionals (in pink) include the availability of evidence-based treatments with established safety and efficacy, and economic factors that affect the availability of services. Cultural contexts influence psychosocial factors (in orange) and reflect individuals' personal history and the community and wider social contexts in which they live. Social factors such as support systems, the implicit biases of healthcare institutions, and stigma (e.g., due to the perceived risk of addiction to opioid analgesics) influence individuals' pain experience and interactions with the health care system. Healthcare policy (in blue) influences the availability of care options (complementary and alternative, non-pharmacological, and biomedical) by determining their affordability, accessibility and acceptability. Different research methodologies can be deployed to investigate the interactions between these variables, including: quantitative data collection through a digital interface (indicated by a screen icon), e.g., analgesic efficacy and safety, cost, and availability of support; participatory action research (community icon), e.g., the study of stigma, community factors, implicit biases or economic factors; and play (theater masks icon) which can provide a flexible frame for studying interactions that are not captured by conventional research methodologies.

Consider the cybernetic model of chronic pain experience and treatment outlined in Figure 2. This diagram represents some of the complex relationships between an individual patient and the health and social care system. A patient in this system is an individual with unique personal experiences, knowledge, values and practices that shape their preferences and choices of treatment. Medical professionals provide care to individuals following current evidence-based practices for safe and efficacious treatment. But economic factors affect the availability of such services. Social and cultural factors such as family and community support systems, illness explanatory models, and expectations affect how an individual interacts with the health care system, which is itself governed by healthcare policy (110). Together this forms a complex system that does not lend itself to the general linear modeling that is common in scientific research.

Bateson's approach suggests identifying pragmatic constraints (e.g., the extent of disability, costs, and accessibility of pharmacological or complementary resources, geographic distance from care systems, and language barriers that present obstacles to service access), and structural constraints (e.g., policies, commercial interests, and capital investment in research and technological innovation) and then examining how variations in cultural factors influence the meanings of individual experiences and behaviors in relation to the social frames and norms that surround an individual. In chronic pain, there are several common conflicts that affect the quality of care and, when these conflicts cannot be escaped because there are no feasible or accessible alternatives, they may constitute double binds. Examples of double-bind situations in relation to chronic pain are listed in Table 1. In the next section, we discuss three important conflicts and double binds that emerge from intersection of qualitative and quantitative approaches to studying and caring for people living with chronic pain.

### Identifying Double Binds

#### Costs of Care vs. Cure

Modern medicine is heavily invested in discovering evidence-based *cures*. However, many CP conditions resist *cure* and instead require long-term *care* (111). Searching for a cure and providing care are both pragmatic responses to the predicament of persistent pain, but in the cultural context of modern medicine, individuals are expected to follow biomedical prescriptions. The imbalances in power arising from professional hierarchies then increase the risk of bias and stigmatization, resulting in blaming patients for lacking mental resolve to be cured, or blaming physicians for their inability to find a cure (112).

In ethnographic studies of chronic pain, anthropologists have shown that shame and stigma are multifaceted sociocultural constructs that are co-created by patients and caregivers (doctors, nurses, families) as well as the larger society (113–115). Providing long-term care is costly, and within societies in which productivity is a virtue, disability—even if socially accepted and accommodated—can be associated with self-reflective shame (116).

Double binds arising from confused messaging around care vs. cure can, in turn, create a pragmatic bias in what kind of treatment approach is acceptable (117). An instance of this conflicted messaging is that while the long-term efficacy of many pharmacological treatments for chronic pain remains uncertain (118–120), complementary, and alternative medicine (CAM) approaches are often dismissed outright by biomedicine as “quack science” (121). Even in a country like Germany—with its 200-year legacy of alternative medicine (122)—only half of physicians surveyed had a positive attitude toward CAMs (123). This is a concern because research has shown that doctors who are prone to dismissing CAMs based on a lack of clinical evidence are also more likely to be dismissive of their CP patients, creating a bias against the research and development required to test the efficacy of these treatments (24, 25). Lack of financial investment to scientifically evaluate what Vos and colleagues call “the orphaned fields of medicine” creates further disparity and inequities in access to care for those who cannot afford the “luxury” of paying for CAMs (124). Strategies to undertake inclusive research to identify cost-effective, efficient, and accessible interventions are needed to overcome persistent biases in knowledge creation and translation to practice. Cost-effective participatory methodologies may help insure inclusivity in research on more effective methods for care.

#### Underlying Mechanisms: Biomedical vs. Psychosocial

Double binds happen when two individuals are involved in an ongoing relationship they cannot exit because it has an important survival value for at least one of them. This can happen between patients and clinicians in health care settings. The poor correlation between extent of tissue damage and level of pain also undermines the credibility of patients' reports of pain. The persistent dualism of Western medical practice makes physicians uncomfortable treating patients who complain of pain with no visible or measurable physical substrate (125, 126). Pain experience is mediated and modulated by a host of psychological processes. However, viewing pain as a condition that is influenced by the mind runs the risk of implying that patients are causing their own problems, whether they are seen as malingering, drug seeking, or suffering from “somatoform disorders” (125, 127, 128). These negative appraisals and biases affect all patients with medically unexplained symptoms but are particularly severe for women and minorities. At the same time, evidence also suggests that the experience of discrimination is associated with increased pain, as found in studies with African Americans (12, 13) and Asian Americans (14). A review of epidemiological studies of pain among North American Indigenous Peoples found that patient-provider interactions generally were rated poorly by patients, who considered healthcare professionals to be uninterested or unsympathetic to their expressions of pain, potentially due to cultural differences in pain communication (129).

While there is a growing acceptance that “pain is whatever patient says it is,” what might be termed a *forensic* approach to diagnosis and clinical assessment aimed at determining the objective truth of “subjective” pain perpetuates implicit biases within the healthcare systems (130, 131). This has a gendered dimension, with women more likely to face dismissive attitudes based on “psychosomatic” attributions of pain (132), further blocking the channels for effective communication of their experiences (133, 134).

Semi-structured interviews with chronic pain patients have found that patients desire care providers who are open-minded, non-judgmental, attentive, and trusting of their expressed needs (135). Yaffe et al. found that trust fostered open communication with care-givers, which can lead to better outcomes (136). Understanding how people narrate (37), and interpret pain across variations in age, gender, culture, or ethnicity requires innovative approaches to data gathering and analysis (137). Beyond quantitative methods, we need to explore experiences more qualitatively and hermeneutically; that is, to learn how to interpret narratives of suffering as they are expressed in conversations, and to examine the ways in which the process of co-construction of meaning unfolds in the course of interactions between patients, clinicians and researchers (37).

#### Cultural Attitudes Toward Pain and Pleasure

Confused messaging can arise from the metaphoric language of “battling” illness which turns the inability to find a cure into failure or defeat, especially in cultural contexts in which competitiveness and winning are virtues (116). This competitiveness may make the notion of “winning” over pain a particular salient trope in Western, individualistic societies, but the determination to endure and contain one's suffering may be motivated by other cultural values that occur in different contexts. A 2018 study found that Indigenous participants in Alberta (Canada) framed their experience with arthritis in terms of “toughing it out”—an approach that allowed them to survive independently of Western medicine. This culturally and historically shaped attitude contributed to their hesitancy to seek timely medical care (138).

There is a substantial literature on ethnocultural differences in coping with pain which reflect differences in styles of emotional expression and communication as well as the meanings of pain and suffering (139–141). For example, a number of factors influence how people from Asian cultures may communicate their pain, such as stoicism (enduring pain or pleasure without expressing it), Buddhism (endurance of pain as a reality of being alive), Confucianism (emphasizing social harmony, and putting society's needs before one's own), social hierarchy status (the physician as holding a position of authority), and, in the case of migrants, language barriers within immigrant communities (142). These cultural variations affect the use of services, modes of clinical presentation and can lead to the false impression that certain populations experience less pain than others.

Cultural differences in the communication of pain and pleasure are based on sensory experiences that are influenced by metaphors that can reshape subsequent experiences (17). This cultural mediation of pain experience points to the importance of a multidisciplinary approach to pain assessment and intervention. Having asserted that access to pain management is a “fundamental human right,” The *Declaration of Montreal* ratified in 2010 by the International Association for the Study of Pain (IASP) (comprising IASP representatives from chapters in 64 countries plus members in 130 countries, as well as members of the community) emphasized the necessity of multidisciplinary pain clinics equipped to address the complex biopsychosocial aspects of CP (143). Yet, despite consensus among professionals and policy makers, the implementation of such programs has been limited because they are costly, and they have remained inaccessible to many in low-income and marginalized communities.

Given the limited availability of multimodal treatment programs and non-pharmacological methods of pain control (144), primary care physicians have focused on medications, including opioids as an expedient approach. This leads to ambiguities and conflicts in communication and treatment negotiation in primary care and other clinical settings (145–148). Miscommunication is also associated with disparities in over- or under-prescription of pain medications to racialized and ethnic minorities (149, 150) that are tied to a lack of attention to social context (151) and to implicit biases that are deeply rooted (152–154). Inconsistencies and lack of trust with regard to permissive or restrictive opiate prescription creates potential double binds in the relationship between healthcare providers and marginalized, racialized, or ethnic minority patients (155).

The double bind in the prescription of opiates arises from the confusing or contradictory messages that opiates have the power to alleviate pain (though inconsistently) (145, 146), but they are 'dangerous' and addictive (148). Moreover, they can also evoke pleasurable feelings and hence, their use may be viewed as a form of self-indulgence and expression of moral weakness (156). Patients who develop opioid dependency struggle when the healthcare system limits access to narcotics (157, 158). In the process, these patients also come to bear the stigma of being labeled as “drug seekers” (159, 160).

To understand the influence of culture and context on how individuals cope with pain and on the delivery of health care, it is important to devise a strategy that accommodates open discussion of both the stoic and hedonic aspects of taking care of one's pain. In what follows, we suggest that modes of exploratory play enabled by digital media can provide a useful component of such a strategy.

## Implementing Play as a Tool for ICT-Based Citizen Science

### Science Museums as Playgrounds for Research

Contemporary science museums, which are designed to provide opportunities for playful exploration of natural phenomena, offer a useful illustration of how we might incorporate play in participatory health research. These museums are set up to allow visitors to explore and interact with exhibits in a flexible way (161). In designing The Museum of Science and Industry in Tampa, Florida, Steier applied Bateson's notion of the flexibility of the frame of play to develop a mode of knowledge generation, by asking: “How do different families interact with the material of an exhibit and how do they interact with each other?” (162). In a science museum, the frame is created by the explicit objective of science communication, the physical location of the installation, the types of science on display, and the specific modes of interaction enabled by media and human docents or facilitators. However, this frame is flexible; it is defined by who chooses to participate, how involved they wish to become, what experiments they choose to explore, and how they engage with and reflect on the activities. Information from these interactions can also inform re-designing the museum to meet the needs of specific groups of users (163).

There are several modes of incorporating play in knowledge creation used in science museums that can be implemented in a digital health research “laboratory.” A virtual simulation of a “science museum” in digital media that simulates the dynamics of exploring exhibits can provide flexible ways for participants to engage and interact with various exhibits on “display.” These exhibits can provide specific modes of playful interaction that serve therapeutic, educational, creative, and experimental objectives. These exhibits can also invite critical reflection on specific issues or questions (e.g., How much does it cost to care for chronic pain? What psychosocial factors exacerbate the burden of pain? How do people from different cultures express and cope with pain? What implicit biases impact on the healthcare system?).

### Play as a Tool for Multi-Modal Data Generation

Citizen Science labs require active participation. In the model of the science museum, this participation takes the form of individuals choosing whether and when to engage with the playful activities or information that are presented in that space. In a play mode, participants may try to challenge the rules to reconfigure the elements, and do so alone or in a group (101–108). Although people often expect to benefit from play as a pleasurable, challenging, or expressive and creative activity that may help them learn new skills, make connections with others, and build relationships, because play occurs within a digitally simulated context, it does not need to be tied to a real-world outcome. As such, play encourages imaginative exploration and provides an opportunity to generate data that can be captured passively (by tracing interactions of users in the digital space) and actively (by inviting users to explicitly record and reflect on their experience and to interact with the arrangement of the space itself to reconfigure and re-create it in ways that suit their own needs and interests).

#### Games

Games are the most widely recognized forms of play. In a bid to capture the motivational aspects of games in non-game contexts, gamification has emerged in the last decade as a popular means of incorporating game-like elements in non-game contexts (164). Even without being designed for specific benefits, playing video games can be helpful for those who cope with mental or behavioral health problems. In a 2018 qualitative study with 20 US Army veterans (165), Colder Carras et al. showed that the participants used video games to manage mood and stress, and as a path to recovery by adaptive coping (e.g., distraction, control, symptom substitution); increasing mastery (confidence, insight, role functioning); and socializing (participation, support, brotherhood). Participants drew meaning from game narratives and characters, and enjoyed exciting or calming gameplay, as well as opportunities to connect, talk, and lead others—benefits that outweighed the problems arising from excessive use of games.

Gamification is increasingly used in rehabilitation and self-care routines to increase adherence and engagement (166–168). The use of game elements in virtual citizen science can bring increased user activity, motivation, and engagement to large-scale scientific projects (169). For example, the video game *Re-Mission* (www.re-mission.net), designed for children and young adults diagnosed with certain types of cancer, aims to educate participants about their illness and ways to manage chemotherapy treatment side-effects. To play the game, players control a virtual nanobot to make strategically effective decisions regarding self-care for side effects of their treatment regimen. Players must complete a mission successfully before moving on to the next level.

There has been considerable research on the analgesic benefits of digital playing for cancer pain (170, 171), burn victims (172–174), pediatric patients (171, 175) and amputees (176), albeit mostly targeting younger players. In pain management, the focus of playing such games is typically on patient education, distraction, or self-management—for example, evoking analgesia through active engagement with VR or digital games (170, 171, 176, 177). When explicitly used for pain control, such interventions can be viewed as *Serious Games*—designed to *benefit* players by training them to improve specific outcome measures (changes in cognitive, physical, and educational variables) against predefined goals (178). Such games serve a double purpose: they can be used both as experimental tools to capture data about patients' learning and behavioral adaptation, and as modes of intervention (if the data suggests efficacy).

#### Storytelling, Art Making, and Interpretation

Creative self-expression activities, such as digital storytelling (179), art making (or performance) or interpretation (180), can provide a context and a vehicle to communicate pain more hermeneutically (37)—that is, to convey not simply the mere fact of pain as a biomedical sensation or index of pathology but also its meaning as an experience mediated by interpretations that emerge from dynamic interactions between patients, clinicians, and researchers. Compared to games, art making and other forms of self-expression may be more flexible media for participatory and qualitative data generation (181). Indeed, internet groups, blogs, and social media already provide flexible tools for self-expression and communication (182, 183).

The language and process of art making are important resources for therapeutic care (184, 185). Reflecting on 3 years of experience with art therapy in palliative care, Bras et al. (186) suggested that medical professionals and patients can adopt the language of art, not only as a means for providing patients therapeutic relief, but also as a means to raise public awareness, and facilitate communication among patients, medical professionals and others involved in decision making about pain. Creative arts therapies have long been offered to individuals with chronic pain (187) to help reduce the psychosocial and emotional burden of chronic illnesses (188).

Digital frameworks can be used for content creation, and dissemination, for example, writing poems and stories, making music, recording sound, and creating images, drawings, videos, and light shows. When shared, the conversations that emerge from encounters with art work can provide language to explore the ways that individuals make sense of experiences that challenge or exceed their usual sensory or cognitive norms (32).

#### Critical Play in Virtual Worlds

Critical play is an important tool for action research. Introduced by Mary Flanagan, *critical play* refers to a genre of interaction that harnesses the pleasure of play in order to communicate and raise awareness about difficult topics, such as social problems arising from implicit biases and stereotypes, and to create transformative models of institutions that overcome these issues in an imaginative space (189, 190). Critical play combines games and art making to provide a space for self-expression, as well as reformatting or re-skinning (changing the surface and the boundaries of a game be it in its aesthetics, dynamics, or mechanics), rewriting and subverting the rules of the play “shifting authority and power relations toward non-hierarchical, participatory exchange” [(189), p. 256]. Critical play also provides opportunities for meta-communication in which frames can be “toyed with” and the rules can be challenged, re-interpreted, and re-written by each person through an iterative process that evaluates their relation to other emerging themes [(189), p. 257].

Unlike goal-driven competitive games, the objectives in critical play are not winning, but rather the imagination, simulation, and communication of new, more suitable, or desirable modes of interaction with self, others, institutions, and the larger social order. For example, *Bill of Health* is a public-health board game in which players grapple with the dynamics of “accountable care” where fee-for-service is replaced by universal healthcare and the players must keep people healthier for an overall price whether they need an expensive surgery, or just smarter health coaching (https://tiltfactor.org/game/bill-of-health/). *RePlay Health* is another example of critical play in a role-playing game (for up to 25 participants) geared toward generating empathy in health policy makers. In this game, players assume the role of patients, care givers and policy makers to explore the healthcare costs through a range of “what if” scenarios (e.g., What if we paid healthcare providers differently? What if our hospitals ran more efficiently? What if everyone only went to the ER when they needed emergency care?) (https://tiltfactor.org/game/replay-health/) (191).

Virtual worlds such as *Second Life* (SL) or *The Sims* are examples of digital laboratories, where players construct an interactive environment with objects, avatars, and rules about how to socialize, trade, make and exhibit art, or disseminate knowledge. Because virtual worlds are highly customizable, participants can create imaginary institutions and places to be inhabited as anonymized or identifiable avatars, depending on the user's preferences. Virtual worlds such as SL provide an opportunity for critical play and simulated decision-making for example in nursing education, where resources or opportunities for creating physical encounters with diverse populations are not available (192–201).

Although digital play may be more limited than in-person play in terms of stimulating deeper conversations (202), the anonymity offered by the virtual world can increase participants' comfort and willingness to engage with research questions, with less concern about social desirability (203). For example, Nosek et al. deployed a pilot weight management intervention program in SL for women with mobility impairments, and demonstrated that while the intervention was feasible and effective it also created new research questions centered on the role of age, gender, and identity as barriers to access to weight loss programs, which could be better facilitated through anonymous avatar self-representations (204).

#### Conversations With Virtual Agents (Chatbots)

Chatbots can be operationalized across three axes: the illness (related to body-mind interactions or lifestyle); the psychology (behavioral monitoring and motivation, affective and cognitive decision-making); and community (connection, personalization, scalability) (205). There is important potential for playful communication with chatbots based on the development of pictorial media in recent years (emoticons, and more recently gif memes) which are creating a new, more universal language of communication (206–209). The usefulness of chatbots as a digital strategy for healthcare has been demonstrated in the detection of suicidal ideation (210), personalized patient education (211, 212), and for qualitative study of patient behaviors (213), or treatment outcomes (214).

Interacting with computerized agents has been found to be conducive to “opening up” (215). Bickmore and colleagues have shown that adding relational skills (empathy, dialogue, non-verbal intimacy, and maintenance of a social and collaborative relationship) to chatbots increased user engagement in a palliative care setting (216). Lucas et al. showed that among active-duty military service members (who were reluctant to disclose their post-deployment health conditions), the anonymity—and, importantly, the rapport—created with a virtual human communicating via online chat was associated with revealing more post-traumatic symptoms than in traditional in-person interviews (217).

An example of a playful clinical chatbot is *Woebot*, a humorous motivational and information delivery about depression. In a study testing *Woebot* with 34 users (who used *Woebot* to become mindful of their mood*)* compared to 36 matched controls (who used the handbook of the National Institutes for Mental Health), Fitzpatrick et al. found that depression symptom scores among *Woebot* users were significantly improved after 2 weeks of use (218).

Chatbots can be deployed for critical play as well. Rice et al. illustrated the value of SL in helping oncology nurses cope with grief in a virtual story-telling environment which did not exist in the personal and professional lives of the grieving nurses (219). Yu and colleagues compared the efficacy of real-time counseling in SL to synchronous face-to-face communication and to asynchronous communication via emails and chatrooms. They found that SL and computerized asynchronous counseling were rated more positively than face-to-face communication for providing anonymity, privacy, diversity of choices for self-representation and environmental personalization, as well as for convenience of time and place of delivery (220).

#### Pitfalls of Play

All media—including playful ICTs—reproduce roles, norms and values derived from cultural contexts (221). Culture also drives *who* plays *what* and *how* (222). This means that any specific approach to play is unlikely to be equally successful in capturing the attention of (and therefore interactions with) diverse individuals. For example, the emphasis on the developmental role of play as an exploratory activity essential for children, can make adults less open to engaging with play unless it is tied to a quantifiable benefit (223, 224). For some, the frame of play may convey a lack of seriousness that undermines the urgency of health problems. In the present instance, the notion of “playing the pain” may itself be construed as a dismissive attitude toward the reality of someone's suffering and undermine efforts to obtain adequate medical care. Thus, working definitions of play (what, how, and why) that respect the priorities and concerns of participants are critical in developing frameworks that engage adults with chronic pain or other health problems.

Competitive games can be engaging for adults both individually and in groups. In *Reality Is Broken: Why Games Make us Better and How they Can Change the World*, Jane McGonigal argues that when we create a simulated environment where players can experiment with various no-cost approaches to solving a problem, we stimulate cooperation among members of the same side to resolve conflicts with opponents (225). In such an approach, activities and interactions are often re-contextualized to include game elements such as points, scores, and rewards that quantify a win.

However, Rey and Bogost criticize the use of play to create virtual commodities and subjects who desire them, because it risks perpetuating a system of social control in which the virtual tokens of reward or achievement metrics replace the real-world resources needed by players (226, 227). The objective of “winning over” rather than “living-with” pain valorizes mastery rather than adaptation (228, 229). As Rilla Khaled has noted, “differentiation and social recognition make sense in mastery-and achievement-focused cultures. […] But in egalitarianism and harmony-oriented cultures […] successful individuals are expected to downplay and de-emphasize their achievements” (p. 308). Even when gamification encourages a community of participants to support and motivate one another, the emphasis on achievement will create hierarchies of success and popularity that can jeopardize community cohesion (230).

A major risk with digitization of play is that it can distort reality without the fully informed awareness and participation of the player. Computational algorithms can offer a narrow space of interactivity to make decisions and take action. This risks turning the play space into a system that forces conformity (231). This goes against the ethics of participatory research. Sicart posits that “the ethical game is not that which evaluates the players' actions according to predetermined moral systems embedded in the game, but that in which the ethics of the game experience and all its elements are reflected on and visible in the game design, in the game experience, and in the game community” (232).

If play is not properly framed, it can also create unintended conflicts—especially if it challenges dominant social norms or moral rules (233, 234). Among the risks of digital media and play that have been identified are stigmatization through social media (235), cyberbullying in massive multiplayer online games (236), and compulsive behavior in Internet gaming disorders (236–238). The challenge in implementing a playground for health research then is developing a mode of interaction that is flexible enough to maximize inclusive participation and leaves room for potential conflicts that arise from divergent views, but provides a frame in which the limits of play are explained and maintained through a shared commitment to creating a safe space for exchange (239).

## Dispora: A Digital Strategy for Play-Oriented Research and Action

### What Is DiSPORA?

DiSPORA is a framework to enable large-scale qualitative research through a digital playground that can be used as a platform for citizen science (specifically, to explore psychosocial and cultural determinants of pain). DiSPORA is grounded in Bateson's cybernetic model, which emphasizes the need to capture the interplay between observable phenomenon and explanatory constructs in order to understand underlying mechanisms that give rise to complex conditions like chronic pain (Figure 1). DiSPORA aims to use play as a way to step outside some of the double-binds that beset chronic pain care (e.g., hard to escape conflicts related to the cost of care vs. cure, the dualism in herent in biomedical vs. psychological explanations, and cultural attitudes toward pain or pleasure—most obviously manifested in the stigmatization of medications associated with addiction). Pain research needs innovative approaches to capture diverse perspectives on these issues. To respond to this need, DiSPORA is designed to serve as a citizen science laboratory for studying the psychosocial aspects of pain experience.

According to Thomas, Scheller and Schroder, a citizen laboratory for social research must offer: (1) a space for social encounters; (2) a frame for communicative practice; (3) a process to initiate social self-understanding; (4) and dynamics that allow participants to engage in and counter public discourse (71). However, pain is also a biomedical condition and, as such, collecting quantitative data is important to conduct analyses that establish the correlations between psychosocial determinants of pain experience and the outcome of interventions. DiSPORA therefore includes a database that can store different data types actively provided by participants (quantitative, narrative, and multimedia) as well as algorithms that passively capture interactions of participants within the playground through the user interface. Ethical data collection requires consent, and DiSPORA includes a procedure to obtain informed consent to participate in pain research that can be tailored to specific projects (see Table 2).

**Table 2:** Research questions, domains of inquiry and potential modes of data collection about factors that influence chronic pain experience and treatment.

**Variables that explain variations in pain experience**	**Domains of inquiry**	**Play-based data collection**			**Traditional data collection**			
		**Game**	**Creative and critical play**	**Chatbot**	**Social network**	**Quantifying instruments**	**Qualitative methods**	**Sensors**
Personal factors	Value-based beliefs		x	x	x		x	
	Behaviors	x			x	x		x
	Affordances	x	x	x	x		x	
	Health states	x				x		x
Economic factors	Affordability		x	x	x		x	
	Accessibility			x	x		x	
	Acceptance		x		x		x	
Social factors	Stigma and implicit bias	x	x	x	x		x	
	Promoting cultural exchange		x		x		x	
	Support systems		x	x	x		x	
Medical factors	Pharmacological interventions					x		x
	Complementary interventions	x	x	x	x	x	x	
	Side Effects			x	x	x	x	x
	Safety and Efficacy				x	x		x

To accommodate self-understanding in a personal and social context, DiSPORA incorporates features from pain diaries that have been used to self-track pain more ecologically, in order to refine the taxonomy of pain disorders, and optimize treatment, based on the psychological and temporal profile of pain experiences (240–242). It includes features such social networking and chatbots to enable collecting communicative data from conversations, narratives, or semi-structured interviews. More importantly, it includes different types of games that may be structured and goal driven (e.g., serious games designed for alleviating pain), or open-ended critical play that allows users to reconfigure and challenge dominant systems of knowledge creation or care in virtual worlds or through creative self-expression. In both cases, the users have the opportunity to critically engage with presumptive discourses (e.g., distraction by game A is analgesic, or care system B is efficient).

As a citizen laboratory, DiSPORA aims to: (1) increase participation of those who may be excluded from research because of geographic location, language skills, lack of recognition of diversity in knowledge and experience, or concern about stigma; (2) enable users to use digital tools (games, surveys, algorithms) to record data from the effects of non-pharmacological interventions that can be delivered or facilitated by digital media (e.g., self-expressive creative arts, serious games, or social networking); and (3) provide opportunities for critical play to reflect on and reconfigure the components and structures of care toward developing a collective action-plan. As such, DiSPORA can be deployed in *N* = 1 studies (e.g., an individual exploring the impact of different gamified therapeutic interventions on their own pain experience), focus-group studies (e.g., a group of researchers and patient partners collaborating through critical playing or storytelling), or hypothesis-driven experiments (e.g., comparing the effects of two interventions to alleviate pain in a large group, while gathering feedback and conducting interviews online).

### Key Dimensions of DiSPORA

#### Framed and Flexible Experimentation Through Play

DiSPORA is framed as a digital playground that can include different tools, such as sensors, questionnaires, or algorithms for computational or statistical data generation and analysis. It can also have different implementations of play to generate different types of data. For example, games can be used for tracing behaviors of users, while interacting with the information and training modules in the game. Virtual worlds can foster creative self-expression or critical play around specific questions (for example by role playing, or construction of new spaces).

In the interpretation of these types of data, the impacts of both frame and flexibility need to be considered. In *Play Matters* (243), Miguel Sicart notes the flexibility of digital forms of play which reflects the ways that they: facilitate the storage and processing of large amounts of data in real-time; have a modular organization that can incorporate diverse modes of data capture such as video, audio, text, sensors (which also can be used to overcome barriers of language); and allow multiple agents to interact with the system in different ways (e.g., in multiplayer games or social networks). Digital forms of play can be deployed on both small and large scales, and personalized to varying extents (e.g., through choice of characters, levels of interaction, and modes of engagement, language, aesthetics). Of course, all digitized spaces are constrained by a more or less rigid frame imposed by computational logic. This makes it possible to do observational research by capturing variations in the ways the people interact with the digital playground and to conduct experiments by introducing small modifications (243, 244). Allowing participants to modify the frame can introduce another level of knowledge generation through active exploration.

**Figure 3:**
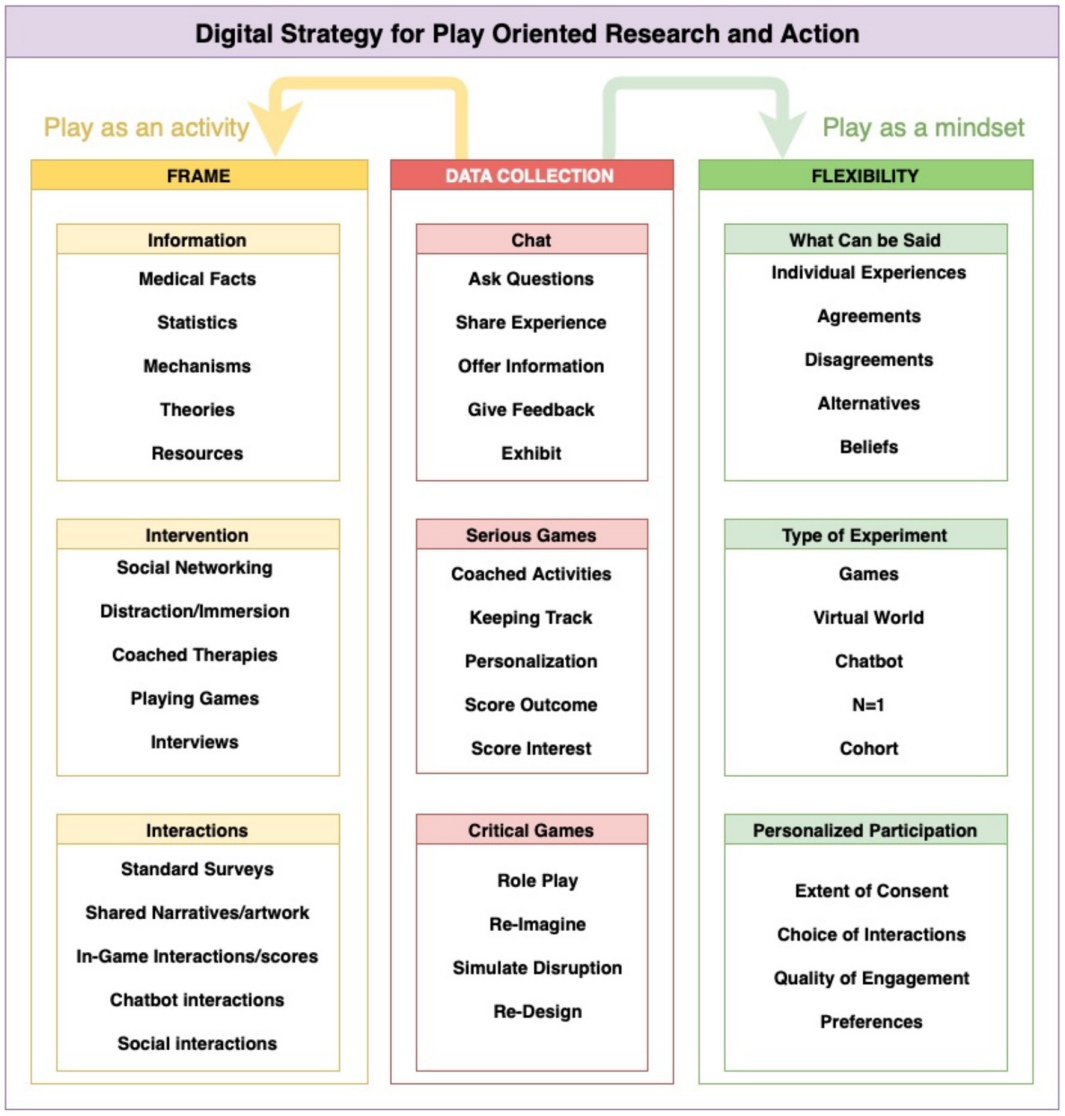
A schematic representation of DiSPORA. Set up similarly to a science museum, the DiSPORA research environment is framed by a set of digitally implemented modules that provide information, offer tools for experimenting with suggested interventions, and capture data from interactions. Play is implemented as a strategy for data collection, encouraging a playful mindset (playing with different ideas) and providing a framework for activities (chatbots, serious games, simulated worlds). The system is designed to be flexible, allowing participants to share ideas or beliefs, decide on the type of experimentation and knowledge generation, select activities, and choose the type and extent of data-sharing they prefer and for which they give explicit consent.

Figure 3 provides a schematic diagram of some of the ways that play can be deployed for data generation (What games do people select to play? With whom? How do they play it?) and a mindset (Why do people select specific games? Do they follow—or break—the rules?). Table 2 provides a summary of the dimensions of experience that can be explored with DiSPORA and multiple modes of data collection, that can be triangulated to improve reliability and find better answers to specific questions (245).

#### Person-Centered but Socially Oriented

An important requirement in the implementation of DiSPORA is to make it *person-centered*—i.e., to respect the validity of the subjective experiences and personal knowledge of each individual. In a meta-analysis of 29 qualitative studies investigating the experiences of patients in patient-centered care models, Winsor et al. found that patients' level of participation was linked to support systems that strengthened their skills for communication of their personal health beliefs, and that recognized their autonomy in self-management (246). In *Coping With Illness Digitally* (247), Stephan Rains reviews evidence to show that meaningful communication and social networking are the most important affordances of ICTs in healthcare. For patients, ICTs can increase *visibility* (making one's self known to others, and vice versa), *availability* (overcoming the barriers of distance, time, and cost), *control* (the ability to limit interactions and exposure), and *reach* (including the potential for selective outreach to relevant groups or individuals). In a *playground* model, each individual's experience is personalized through their decision to engage in certain activities, but the activities occur in a shared virtual space. This shared space offers opportunities to observe and interact with others, co-learn, offer feedback, or share narratives. The modular structure of DiSPORA makes it possible for participants to suggest changes that will improve the playground to better meet their needs.

#### Inclusive and Tolerant of Conflicting Views

A goal in designing DiSPORA is to contribute to equity in health research and practice by enabling participants from diverse cultural backgrounds to contribute knowledge based on their health experiences and perspectives. Structural inequities in society have led to systematic biases in research participation, design, sampling, and measurement. These include biases in what forms of knowledge production and authority are recognized, which can result in what Fricker has termed *epistemic injustice* (27, 97, 98). Therefore, it is expected that in a social and interactive space that invites feedback and personal narrative, conflicts will emerge. However, as Bergold and Thomas have argued, creating a situation that tolerates conflictual views also creates conditions for valuable self-reflexivity and negotiation to advance research (248).

Similarly to our proposal, Thomas et al. have argued that meta-communication provides a path forward in resolving conflicts in participatory citizen research projects (71). This requires that the users of DiSPORA are made aware of the risks of such conflicts, and consent to play to communicate (rather than to receive pain “therapy” or “find a winning solution”). Therefore, to ensure self-reflexive mediation of conflicts, DiSPORA needs to have professionally facilitated or moderated dialogues to ensure that stakeholders engage with one another without a vocal minority or the forceful majority silencing any participant's unique experiences and perspectives.

### A Prototype of DiSPORA

**Figure 4:**
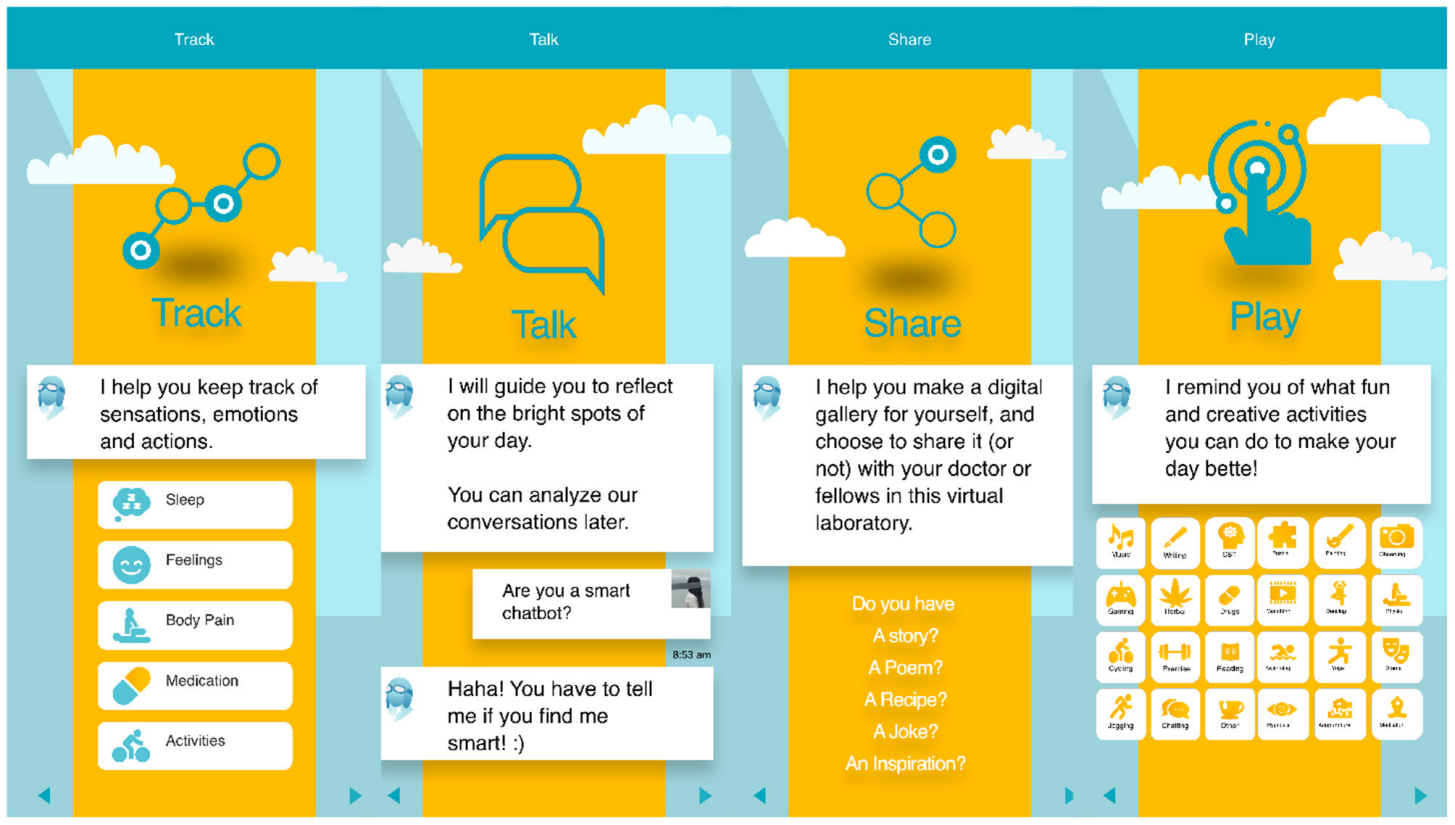
Features of PlaythePain App. The app allows uses to track their pain experience in ways similar to conventional pain diaries. Data-generation via play is supported by features including: *Talk*, where an interactive chatbot encourages conversations about various topics which the player selects; *Share*, a social-media like forum for sharing art and ideas; and *Play*, a catalog of various playful activities that are coached through third-party applications, either suggested by researchers or introduced to the forum by participants.

In line with DiSPORA, as proof of principle, we have recently developed an app (PlaythePain.com) to examine the affordances of playful, creative and social activities in a digital citizen laboratory (86). The app is designed to serve as a participatory citizen science laboratory, and respect individual differences in focus, interests, modes of self-evaluation, self-expression, and self-care. As such, a range of customization features are available to allow users to select what to play, what data to share, and with whom. This prototype has been launched in both English and French (and, with the exception of the chatbot, it strives to communicate visually; see Figure 4). In this pilot project, we have included five features:

- **Talk**: An chatbot that encourages reflection and allows semi-structured qualitative data collection about various aspects of daily experiences;
- **Track**: A digital pain diary to record information about sleep, activities, feelings, and medication;
- **Share**: A restricted social network that allows users to create a gallery of their stories, artwork, and videos about various activities or experiences that they wish to share with other participants;
- **Play**: A library of different mHealth-mediated interventions (diet, exercise, brain training), as well as games. A default set is available initially, but users have the ability to add their own games and linked apps as well;
- **Report**: A comprehensive report of all data collected within the app, transparently available to the user which can be shared only if they grant access to a designated research-partner.

Individuals can join this platform anonymously (by registering via email) and after signing the terms and conditions that emphasize the need to adhere to social norms and behave courteously in socialization with other users. Users are unrestricted in the content of their conversation with chatbots, or in sharing private stories which can be mined in the future for the purpose of research. In future work, we plan to test and integrate this version of *Play the Pain* in our local neuroinformatics ecosystems (249).

### How DiSPORA Could Serve as a Tool for Participatory Health Research

Medical care faces the challenge of understanding the complex interactions between brain, person, and environment that give rise to problems like chronic pain as well as the processes of healing and recovery (137, 250). Precision medicine and psychiatry aim to replace one-size-fits-all models in healthcare with more tailored, personalized approaches that consider biology, behavior and environmental context (251). A growing body of evidence demonstrates that environmental and psychosocial factors that affect brain plasticity also impact on physical health throughout the lifespan (252). This points to the need for a multilevel (bio-psycho-social) framework that encompasses the physiological, psychological, and social processes that are constitutive of experience in health and illness (29, 30). To date, however, precision medicine has focused primarily on biological factors which are assessed with laboratory measures.

Patients, patient-advocacy groups, nurses and social workers, and qualitative public health researchers have played an important role in shifting healthcare from a purely medical model to one that is person-centered (29, 30), involving patients in clinical decision-making, and self-management (135). Considering the patient as a person in a social context or life world can help address issues of diversity and representativeness in health research, including differences associated with gender, ethnicity, education, and social class and their intersections (78–80, 137).

One way to address the limitations of the narrow biological approach is to democratize knowledge-creation by empowering patients to take charge of their evaluation and the development and delivery of interventions informed by their lived experience (74–76). This can be achieved through cooperative participation of various stakeholders in a process that gives everyone, especially those who are marginalized, a voice to contribute to research and policy-making through knowledge-sharing and co-learning (40, 77). The value of such patient empowerment approaches has long been recognized in public health (77), particularly in efforts to address the social determinants of health (78, 79).

However, methodological challenges related to rigorous data gathering (38), communication (39), and inclusivity (40) limit the use of qualitative research practices in large-scale health research. In the section above on *Integrating Context and Experience in Pain Research* we suggested some ways in which ICTs can benefit such research, and in section *The Promise of Information and Communication Technologies (ICT) for Integrative Pain Research*, we listed the shortcomings of existing implementations of ICT in health research. DiSPORA proposes to overcome some of these limitations by creating a framework for capturing participants' perspectives through a scalable framework that takes advantage of the affordances of play as a meta-communication strategy (32, 205, 225, 243, 247) in a citizen science participatory research laboratory (47, 48, 53, 71, 169). Of course, in itself, the use of DiSPORA does not establish the conditions necessary for participatory research. Achieving true participation requires setting up and maintaining essential protocols and structures of governance for processes of partnership and collaboration that are well-described in the literature on participatory health research (69–73).

## Conclusion

### Main Arguments

Medical researchers are increasingly aware of the need for qualitative and participatory research to inform policy and clinical practice. To help address this need, our interdisciplinary team of clinicians, social scientists, bioethicists, designers, and computer scientists developed an innovative ICT framework for knowledge generation in chronic-pain research.

Examining chronic pain from the perspective of the Neuromatrix Pain Theory (9), Pain Communication Theory (15), Cultural Somatization Theory (87), and person-centered action-research in chronic pain (23, 32, 37), we asked whether and how ICTs can improve research that can integrate these theories. We adapted Bateson's cybernetic approach to studying mind-body processes in ecosocial context. In Bateson's cybernetic model, the links between the observable phenomena and underlying mechanisms are mediated by culturally contextualized explanatory factors. We also identified some double binds (inescapable tensions or conflicts) that need to be addressed in pain research involving issues that are structural (investing in care vs. cure), pragmatic (investing in biomedical vs. psychosocial), and cultural (moralizing attitudes toward pain and pleasure).

We adapted play (a mode of communication, simulated action or performance, and creative disruption) as a framed but flexible research tool for meta-communication and for recursive knowledge generation about pragmatic, structural, and cultural conflicts in pain research. To clarify the potential uses of play in research, we reviewed evidence suggesting that *playing* games, role-playing in virtual worlds, engaging in creative arts and self-expression (e.g., through talking to chatbots or social networking) offer opportunities for passive and active data generation.

Finally, we proposed a model for operationalizing play in citizen science research we call DiSPORA. Drawing inspiration from the design of science museums, DiSPORA provides a digital playground that includes interactive information delivery, games, social networks, virtual chatbots, and simulated virtual worlds. Participant interactions with DiSPORA are facilitated to ensure that data is captured in ways that are safe and inclusive. The key elements of DiSPORA are: a clear but flexibile frame; person-centered but enabling sociability; and tolerance for diversity and inclusivity. A simple prototype app has been constructed to illustrate how this citizen lab could be implemented.

### Limitations and Next Steps

DiSPORA is a proposal for how to design an ICT framework to engage patients as active participants in studying diverse psychosocial questions that arise in relation to their healthcare needs. However, in itself, a framework does not ensure the social relationships, ethical commitments, or organizational structures and procedures that are necessary for participatory research and action.

Although we think that the modular design of DiSPORA can serve as a flexible frame for citizen research, the fact that DiSPORA is governed by its computational logic may limit its ability to capture important dimensions of experience not built into its algorithms. The social networking and collaboration that can be enabled by DiSPORA might allow stakeholders to tailor it to their own research questions and needs. However, recent global experiences with social media raise concerns about the risks of disseminating misinformation, or exacerbating bias and stigma through ingroup/outgroup dynamics. In addition, important ethical issues related to privacy, individual recognition, and data sovereignty remain to be addressed (253). These are important research questions that can be explored in the collaborative refinement of DiSPORA applications through an agile recursive design process (254).

Finally, reliance on digital media and play to engage people in exploring the quality, parameters, and modulators of their pain experience is based on the assumption that play can afford people some distance from the suffering associated with their health problem, while mobilizing both coping strategies and social connection to others. Whether these characteristics of play can be maintained through interaction with a digital citizen lab remains to be tested. However, our own recursive learning process in developing this concept and feedback from peer reviewers have provided encouragement and valuable lessons about reaching consensus on the basic requirements for research that respects individual and diverse ways of knowing and knowledge generation.

## Author Contributions

NK-M, TG, and MR conceptualized the Play the Pain project. NK-M, EH, and SW conducted the research. NK-M, EH, SW, LK, RK, BS, ML, MN, and RH contributed to the analysis and co-wrote the paper. AD and JT-B provided expert advice on clinical and community practices of care, respectively. All authors contributed to the article and approved the submitted version.

## Funding

This research was funded by a grant from the Quebec Research Funds to NK-M (FRQSC-AUDACE), additionally supported by MCIN (McGill University), Milieux Institute for Art, Culture and Technology, PERFORM Center (Concordia University), and the Healthy Brains for Healthy Lives Program (CFREF, McGill University).

## Conflict of Interest

The authors declare that the research was conducted in the absence of any commercial or financial relationships that could be construed as a potential conflict of interest.

## Publisher's Note

All claims expressed in this article are solely those of the authors and do not necessarily represent those of their affiliated organizations, or those of the publisher, the editors and the reviewers. Any product that may be evaluated in this article, or claim that may be made by its manufacturer, is not guaranteed or endorsed by the publisher.
